# Editorial: Head and neck cancer in the elderly

**DOI:** 10.3389/fonc.2023.1218274

**Published:** 2023-06-22

**Authors:** Petr Szturz, Jan B. Vermorken

**Affiliations:** ^1^ Department of Oncology, University of Lausanne (UNIL) and Lausanne University Hospital (CHUV), Lausanne, Switzerland; ^2^ Department of Medical Oncology, Antwerp University Hospital, Edegem, Belgium; ^3^ Faculty of Medicine and Health Sciences, University of Antwerp, Antwerp, Belgium

**Keywords:** geriatric medicine, epidemiology, quality of life, systemic treatment, radiotherapy, palliative care, salivary gland cancers, nasopharyngeal carcinoma

In oncology, geriatric medicine represents a topic with contrasting perspectives. On the one hand, it is familiar to many of practicing physicians, on the other hand, high-level evidence to guide our treatment decisions for this patient population is lacking. Although accumulating data show that treatment benefit can be retained with advancing age in fit patients, it is not unusual that when facing an older adult, even in good health, a less intensive approach is prioritized to avoid excessive toxicity (1). While the importance of increasing awareness and generating prospective clinical evidence regarding these aspects is self-explanatory, little has changed over the past decades. Elderly patients are still underrepresented in clinical trials, often receive suboptimal therapy based only on the difference in chronological age, and according to a recent survey involving almost 400 US community-practice oncologists, geriatric assessment tools have not been adopted by the majority of them (2, 3). The reasons for that are obvious. Older adults often suffer from multiple comorbidities making them vulnerable or frail and thus increasing their odds of non-cancer related deaths; enrolment of such a patient population in clinical trials is challenging and puts additional strains on quality of trial design and potentially also on costs; and geriatric assessment tools have been perceived as difficult to implement in clinical practice and/or to have limited added value for patient management.

These shortcomings seem to be even more pronounced in head and neck cancer (HNC) patients. Specific anatomic characteristics in the head and neck area contribute to the complexity of radiotherapy planning, which may be associated with further challenges regarding toxicity in senior persons. Moreover, well-known carcinogenic risk factors including alcohol consumption and tobacco use lead to collateral damages in terms of multifaceted comorbidities, especially after a long-term exposure being typical for elderly people. Finally, any deformities in a visually exposed region as is the area of the head and neck may have far-reaching impact on quality of life in this patient population with specific considerations in older adults as explained below. This background motivated conceptualization of the present article collection focusing on different aspects of anticancer care in elderly HNC patients and discussing the evolving epidemiological landscape as well. In this respect, absolute and relative numbers of HNC cases in the elderly have been steadily rising, particularly in high-income countries where almost 50% of newly diagnosed HNC patients are older than 65 years of age. Nevertheless, their 5-year overall survival rates still fall behind outcomes seen in younger patients in European countries, ranging between 60% in salivary gland and laryngeal cancers to only 22% in hypopharyngeal cancers (Gatta et al.). Although a substantial proportion of deaths in the elderly population is due to non-cancer related competing mortality, wide-reaching improvements in anticancer management of elderly people is required with some aspects being covered in this article collection.

Representing the mainstay of anticancer management in HNC patients, radiotherapy has been used across different clinical settings and patient populations. As mentioned above, the heterogeneity of elderly individuals can lead to treatment modifications towards less intensive schedules. Therefore, good understanding of current clinical practice patterns is welcome in order to identify areas necessitating better targeting in clinical trials, treatment guidelines, and awareness programs. Haehl et al. conducted a tri-national patterns-of-care survey among national societies of radiation oncology in German speaking countries (Germany, Austria, and Switzerland). The investigators received answers from 132 respondents with only half of them declaring that radiotherapy target volumes corresponded to current treatment guidelines irrespective of the patient age, while 28% reported to adapt target volume definition in dependency of age and comorbidities and 22% in dependency of the tumour stage. Although the respondents administered concurrent chemotherapy regardless of chronological age, they were far more restrictive in terms of altered fractionation regimens, including hypofractionation. The latter schedule defined by using at least 2.2 Gy fractions was a subject of a systematic review by Piras et al. aiming to find out whether hypofractionation is a safe and feasible option in patients of 65 years of age or older. Based on data from 17 included papers, the authors conclude that a dose of 55 Gy delivered in 20 fractions represents a viable alternative to the standard fractionated radiotherapy, although the choice of a specific regimen needs to be individualized.

Another topic that has recently been brought to the spotlight of researchers concerns rare diagnoses. Interestingly, they share some characteristics attributed to the elderly cancer patients, namely paucity of high-level evidence and the resulting uncertainty about appropriate management. Typical examples are salivary gland cancers and nasopharyngeal carcinoma, particularly in non-endemic regions. Now, what happens if the two categories, elderly population and rare diseases, are merged? You will find possible answers in two contributions to our collection. In a comprehensive review about management of salivary gland cancers, Colombo et al. point out some of the common practice patterns in elderly patients and relate them to recommended procedures including but not limited to geriatric assessment and screening tests. Elderly patients with salivary gland cancers are diagnosed more frequently at advanced stages with a higher proportion of more aggressive (high-grade) histotypes and lower 5-year overall survival rates per disease stage in comparison with their younger counterparts. Interestingly, it seems that chronological age plays a less important role in elderly patients with salivary gland cancers than with other types of HNC when it comes to surgery, probably due to anatomic differences influencing feasibility of resection and a generally lower sensitivity of salivary gland cancers to radiation and systemic therapies. The authors present a very workable algorithm for the clinical assessment and treatment and give a clear summary of cytotoxic and non-cytotoxic systemic therapy options readapted to the elderly population.

The principles of geriatric medicine based on biological rather than chronological age has been brought forward also by Chan et al. in their review article about management of elderly people with nasopharyngeal carcinoma. Involvement of this anatomic site leads to specific challenges in delivery of local treatment owing to its complex geometry and the fact that older persons are more likely to develop treatment-related toxicities. In addition, nasopharyngeal carcinoma affects only about 10-15% of patients of 70 years of age or older, i.e., about a half of what is observed in HNC of other subsites (oral cavity, oropharynx, larynx, and hypopharynx), and this raises a further barrier to data availability (Chan et al., 4). Unlike salivary gland cancers, the stage distribution pattern of nasopharyngeal carcinoma does not depend on age, and disappointingly, their management shares a common feature with other HNC types in that often less aggressive treatment is used in the elderly. In response to the latter observation, the authors present an algorithm for management of these patients according to their biological age and the clinical setting.

The third area covered in this Research Topic has been addressed by Schrijvers and Charlton reporting on palliative care and end-of-life issues and by McDowell et al. describing health-related quality of life, psychosocial distress, and unmet needs in older patients with HNC. Symptoms including fatigue, lack of energy, malnutrition, pain with associated signs of loco-regional or distant tumour progressions, psychosocial problems, and spiritual issues linked to end-of-life decisions are typically encompassed in the area of palliative medicine but also demonstrate that this discipline has evolved to holistic care based on partnership between doctor and patient and supported by informal carers involving family and friends. In this regard, continuity is the most important aspect that we need to consider when dealing with palliative and end-of-life care (Schrijvers and Charlton). Due to the increasing prevalence of frailty and vulnerability with advancing age, elderly people are prone to acquire such a complex image of HNC. Nevertheless, fit older adults self-report greater resilience in health-related quality of life and psychosocial outcomes than younger individuals. It is also of note that judging by data from studies about variations in unmet needs by age in HNC, either no difference was observed or a lower burden was seen in senior persons who also stated less unmet needs for individual domains such as sexual, psychological, emotional, and spiritual needs (McDowell et al.).

In conclusion, we believe that this Research Topic will provide interested readers with a comprehensive overview of HNC in the elderly ranging from practice-oriented topics over areas touching up on rare entities all the way to important considerations gaining attention only in recent years, including psychosocial aspects and unmet needs. Altogether, the ever-growing population of older cancer patients requires individualized measures adapted to their biological and not chronological age. Undertreatment, underrepresentation in clinical trials, and disregarding specific patient requirements represent the major issues in daily practice we will have to address in the near future to move forward.

## Author contributions

All authors listed have made a substantial, direct, and intellectual contribution to the work and approved it for publication.
